# Assessment of lower urinary tract symptoms and their impact on quality of life following radiation therapy for bladder cancer

**DOI:** 10.14440/bladder.2024.0046

**Published:** 2025-02-21

**Authors:** Josephine Hyldgaard, Anne Birgitte Als, Benedicte Ulhøi, Jørgen Jensen, Charlotte Graugaard-Jensen

**Affiliations:** Department of Urology, Aarhus University Hospital, Aarhus 8200, Denmark

**Keywords:** Bladder cancer, Radiation therapy, Radiation cystitis, Quality of life, Late effects

## Abstract

**Background:**

Radiation therapy is a common treatment modality for muscle-invasive bladder cancer (BC). Unfortunately, approximately 5 – 10% of treated patients suffer from long-term complications, such as radiation cystitis. While radiation cystitis initially causes urinary symptoms, it can progress to chronic late effects over time. This treatment may have significant physical and psychological implications for cancer survivors.

**Methods:**

This prospective, observational multicenter study was conducted in Denmark from 2020 to 2023. Male patients undergoing radiation treatment for BC were included and stratified into two groups based on radiation strategy: 64 Gy/32 fractions versus 36 Gy/6 fractions. Patients completed baseline and follow-up questionnaires assessing lower urinary tract symptoms and quality of life (QoL).

**Results:**

A total of 26 patients were enrolled into the study, with three patients withdrawing consent. Eleven patients were assigned to the 64 Gy/32 fraction group and 12 to the 36 Gy/6 fraction group. At baseline, patients receiving 64 Gy/32 fraction reported a lower impact on QoL, with a median (interquartile range [IQR]) score of 2 (24) compared to 20 (27) in the 36 Gy/6 fraction group. At the 4-month follow-up, the median (IQR) total QoL score was 25 (27) in the 64 Gy 32 fraction group and 6 (14) in the 36 Gy/6 fraction group. At 12 months, the scores were 16 (35) and 3 (3), respectively.

**Conclusion:**

These findings suggest that the total radiation dose has a greater impact on urinary symptoms and QoL than the fractionation strategy. However, due to the low inclusion rate, these findings should be interpreted with caution.

## 1. Introduction

Bladder cancer (BC) is the most common cancer of the urinary tract system. It is divided into muscle-invasive BC (MIBC) and non-MIBC.^1^ The primary treatment for MIBC is radical cystectomy, with or without neoadjuvant chemotherapy. However, some patients may be ineligible for surgery or may opt for a bladder-sparring approach, and they undergo trimodality treatment. This treatment combines transurethral resection of the bladder tumor (TURBT), external beam radiation therapy (RT), and chemotherapy.^2^,^3^ In addition, some patients who are ineligible for surgery received RT as monotherapy following TURBT.^4^ According to the Global Cancer Observatory, the incidence rate of BC in Europe stands at roughly 12%.^5^ In Denmark, 912 patients were diagnosed with MIBC in 2023, of whom 64 received curative-intent RT.^6^

The Danish National BC Guidelines recommend radical cystectomy as the first-line treatment for patients who are eligible for extensive surgery and urinary diversion. However, for selected patients with sufficient bladder function, trimodality treatment is considered an equivalent to cystectomy.^7^

RT is considered an effective treatment modality due to its ability to disrupt DNA synthesis, thereby inhibiting the proliferation of rapidly dividing cancer cells. However, its effects are not tumor-specific, and normal surrounding tissues are also affected, potentially leading to treatment-related complications. One such complication is radiation cystitis, which can develop following RT to the pelvic area, such as treatments for prostate, bladder, or gynecologic cancers.^8^,^9^

Acute radiation cystitis (ARC) typically develops within 6 months of RT and is characterized by acute inflammation and edema of the bladder mucosa. This process impairs urothelial regeneration, thereby compromising the protective barrier between urine and the bladder epithelium. Consequently, the bladder becomes more susceptible to trauma and infection.^8^,^10^ Hematuria is the most common symptom, often accompanied by pain and dysuria, as well as irritative voiding symptoms such as frequency, urgency, and incontinence, all of them contributing to a significant decline in patients’ quality of life (QoL).^8^ Despite these symptoms, ARC is usually self-limiting.^10^,^11^

**Figure 1:**
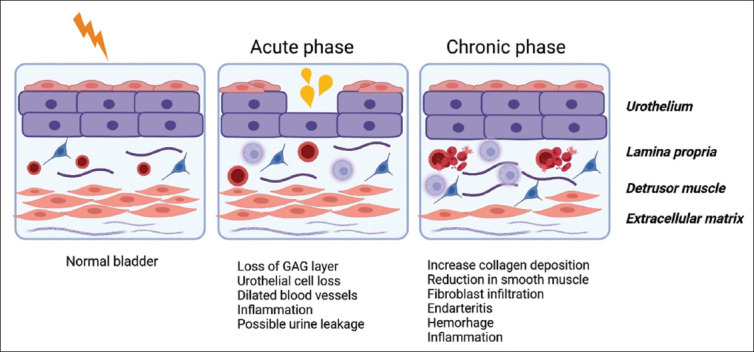
The acute and chronic phases of radiation cystitis during radiation therapy. Abbreviation: GAG: Glycosaminoglycan.

Chronic radiation cystitis (CRC) develops more than 6 months after RT and afflicts 5 – 10% of patients, with an average onset time of 31.8 months post-treatment.^12^ This delayed-onset cystitis results from progressive damage to bladder tissue due to fibrosis of the vascular intima, resulting in vessel obliteration, submucosal and muscular fibrosis, and urothelial atrophy. The emergence of telangiectasia further increases the risk of recurrent hematuria, the hallmark symptom of CRC.^8^,^13^ In later stages, fibrosis leads to a reduction in bladder capacity and compliance, increasing the risk of mucosal ulceration, fistulization, and spontaneous bladder perforation. In addition, reduced bladder capacity and compliance heighten the risk of kidney damage.^9^ The progression of radiation cystitis is depicted in Figure 1.

The risk of long-term complications appears to be associated with radiation protocols and is more common in men than in women.^12^ Several factors influence the likelihood of complications, including bladder surface area exposed to radiation, dose per fraction (a dose exceeding 2 Gy/fraction increases the risk of sequelae), and total radiation dose (toxicity risk is significantly higher when the overall dose is over 60 Gy to the bladder).^14^

At present, no curative treatment is available for CRC. As a result, the radiation dose used in cancer treatment is limited by the potential risk of late effects.^9^ A current RT strategy for treating locally advanced BC is 64 Gy in 32 fractions.^15^ However, for patients who are considered unsuitable for radical treatment, an RT regimen of 36 Gy in 6 fractions is typically chosen.^16^

This observational study aimed to describe the side effects of RT for BC as reported by patients and to evaluate how these changes affect the QoL in patients treated with two different RT strategies.

## 2. Methods

### 2.1. Study design

This prospective, observational multicenter study was conducted in Denmark from 2020 to 2023. Male patients were eligible for inclusion if (1) they had been diagnosed as having T2 – T4a, N0, M0 BC, (2) were scheduled for RT, and (3) provided informed consent for participation. Due to sex-based differences in BC incidence, diagnosis, and treatment outcomes, only male patients were included in the cohort. Patients were eligible regardless of whether they had a preoperative urinary catheter. Patients were stratified in terms of the radiation protocol, receiving either 64 Gy in 32 fractions or 36 Gy in 6 fractions. Exclusion criteria included (1) any other BC stage, (2) refusal to participate, or (3) inability/reluctance to provide informed consent.

Patients were recruited from four hospitals based in Denmark: Aarhus University Hospital, Aalborg University Hospital, Odense University Hospital, and Regional Hospital Herlev/Gentofte.

The study was approved by the Danish Ethical Committee (no. 1-10-71-272-19), and all patients were informed of their right to withdraw consent or decline participation at any given time. Data collection complied with the Danish Data Protection Regulation and the Danish Data Protection Agency’s legal framework. All patient data were securely stored in a REDCap^™^ database. The study was registered on ClinicalTrials.gov (NCT04282876) and endorsed by the Danish BC Group (DaBlaCa-18).

Both treatment and follow-up procedures adhered to national guidelines as prescribed by the urology departments at participating hospitals.

### 2.2. Standard follow-up

The standard follow-up protocol for patients undergoing RT included cystoscopy with biopsy performed under general anesthesia 3 months after the final radiation fraction to evaluate treatment response. In addition, a computed tomography (CT) scan was performed to rule out distant metastasis. If the CT scan or biopsy indicated progression or recurrence of BC, patients were managed by their designated physician at the participating hospital in accordance with national guidelines and were excluded from the study. If no residual disease was detected, patients continued follow-up with regular outpatient cystoscopy every 4 months for 2 years, following national guidelines.

### 2.3. Patient-reported questionnaires and data collection

Upon inclusion, patients were asked to complete validated paper-based questionnaires assessing bladder symptoms against the international consultation on incontinence questionnaire male lower urinary tract symptoms (ICIQ-MLUTS) (Appendix) and the potential impact of these symptoms on QoL. Furthermore, patients were instructed to complete a paper-based frequency-volume chart. At the initiation of RT, patients were further instructed to maintain a paper-based bladder function diary at home for each week of RT, recording information on cystitis symptoms, hematuria, daily voiding frequency, nightly voiding frequency, involuntary voiding episodes, dysuria, and catheter use. Once per week during treatment, the primary investigator conducted telephone follow-ups to evaluate patient-reported symptoms and record them in the online database.

Furthermore, patients were asked to complete the same paper questionnaires at two follow-up time points: 4 months after the final RT fraction and 12 months post-treatment. The 4-month follow-up was chosen to allow sufficient time after RT and the control cystoscopy with biopsy, while the 12-month follow-up was selected to assess potential late effects and fibrosis development. The questionnaires were mailed to the patients for completion.

### 2.4. Statistical considerations

For continuous variables, patient characteristics were presented as the median and interquartile range (IQR). For categorical variables, results were expressed as counts (*n*) and percentages (%). Due to the small sample size in each category, data from patient diaries were reported only as counts (*n*). Data from patient questionnaires were reported as median and IQR. This study was purely of a descriptive nature, without any inferential statistical analysis. Data analysis was performed using R studio.

## 3. Results

### 3.1. Patient enrollment and group allocation

**Figure 2:**
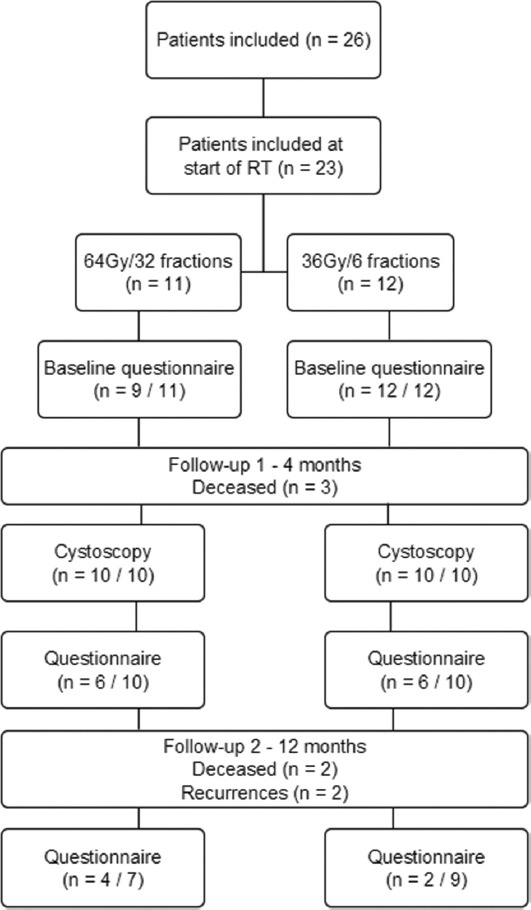
Flowchart of patient recruitment and follow-up processes Abbreviation: RT: Radiation therapy.

During the study period, a total of 26 patients provided informed consent for participation. However, three patients withdrew consent before initiating RT. Among the remaining 23 patients, 11 patients were assigned to the 64 Gy/32 fraction RT protocol, while 12 to the 36 Gy/6 fraction protocol as shown in Figure 2.

As presented in Table 1, patients receiving 64 Gy were generally younger and more likely to have received neoadjuvant chemotherapy compared to those in the 36 Gy/6 fraction group. In addition, patients in the 36 Gy/6 fraction group were more likely to be former smokers.

**Table 1:** Baseline characteristics of patients included

Characteristics	64 Gy/32 fraction group (*n*=11)	36 Gy/6 fraction group (*n*=12)
Age, median (IQR)	78 (10)	85 (6)
BMI, median (IQR)	27.7 (4.3)	26.4 (7)
CCI, *n* (%)		
0	1 (9.1)	4 (33.0)
1 – 2	8 (73.0)	7 (55.0)
>2	2 (18.1)	1 (12.0)
Smoking		
Current	5 (45.5)	2 (17)
Never	2 (18.1)	0 (0.0)
Previously	4 (36.4)	10 (83)
Diabetes		
Type I	0 (0.0)	0 (0.0)
Type II	5 (45.5)	1 (8.3)
Back problems	3 (27.3)	4 (33.0)
Urinary catheter		
No	7 (64.0)	8 (67.0)
Yes	4 (36.0)	4 (33.0)
Neoadjuvant chemotherapy	3 (27.3)	0 (0.0)
Bladder irrigation	1 (9.1)	1 (8.3)
PC diagnosis	1 (9.1)	1 (8.3)

Abbreviations: BMI: Body mass index; CCI: Charlson comorbidity index; IQR: Interquartile range; *n*: Number; PC: Prostate cancer.

### 3.2. Follow-up protocol and specimen analysis

Following RT, a total of 13 patients underwent cystoscopy with biopsy, 12 under general anesthesia, and one through flexible cystoscopy. An additional seven patients received flexible cystoscopy without biopsy, while the remaining three patients passed away before follow-up. During follow-up, two recurrences were detected in the 64 Gy/32 fraction group, as shown in Table 2.

**Table 2:** Distribution of participants during follow-up

Follow-up protocol	64 Gy/32 fraction group (*n*=11)	36 Gy/6 fraction group (*n*=12)
Cystoscopy+biopsy, *n*	10	3
Flex cystoscopy without biopsy, *n*	0	7
Recurrence, *n*	2	0
Deceased, *n*	1	2

Abbreviations: IQR: Interquartile range; *n*: Number.

### 3.3. Questionnaires and symptom diaries

Baseline questionnaire results (Table 3) were similar between the two groups, with a total lower urinary tract symptom (LUTS) score of 12 (13) in the 64 Gy/32 fraction group and 11 (15) in the 36 Gy/6 fraction group. Both groups had higher scores for voiding symptoms compared to storage symptoms. The median QoL score for storage symptoms was lower in the 64 Gy/32 fraction group, with a median of 1 (12), compared to 10 (14) in the 36 Gy/6 fraction group.

**Table 3:** Baseline questionnaires

Baseline	64 Gy/32 fraction group (*n*=9/11)	36 Gy/6 fraction group (*n*=12/12)
MLUTS total, median (IQR)	12 (13)	11 (15)
Voiding symptoms, median (IQR)	8 (5)	7 (6)
Storage symptoms, median (IQR)	4 (3)	4 (9)
QoL voiding, median (IQR)	4 (16)	2 (14)
QoL storage, median (IQR)	1 (12)	10 (14)
QoL total, median (IQR)	2 (24)	20 (27)

Abbreviations: IQR: Interquartile range; MLUTS: Male lower urinary tract symptom score; *n*: Number; QoL: Quality of life.

At the 4-month follow-up, 12 out of 20 patients completed the questionnaire, respondents being evenly distributed across both groups (Table 4). Total LUTS scores remained comparable, with a median of 11 (9) in the 64 Gy/32 fraction group and 9 (5) in the 36 Gy/6 fraction group. However, QoL scores increased in the 64 Gy/32 fraction group, with a median total QoL score of 27 (25), whereas the 36 Gy/6 fraction group had a median total QoL score of 6 (14).

**Table 4:** Follow-up questionnaires (4 months)

Follow-up 4 months	64 Gy/32 fraction group (*n*=6/10)	36 Gy/6 fraction group (*n*=6/10)
MLUTS total, median (IQR)	11 (9)	9 (5)
Voiding symptoms, median (IQR)	8 (5)	6 (5)
Storage symptoms, median (IQR)	5 (4)	4 (1)
	16 (24)	0 (6)
QoL storage, median (IQR)	11 (14)	6 (8)
QoL total, median (IQR)	27 (25)	6 (14)

Abbreviations: IQR: Interquartile range; MLUTS: Male lower urinary tract symptom score; *n*: Number; QoL: Quality of life.

At the 12-month follow-up, four patients from the 64Gy/32 fraction group and two from the 36Gy/6 fraction group completed the questionnaire (Table 5). The median total LUTS score for the 64 Gy/32 fraction group increased to 20 (6), primarily due to an increase in storage symptom scores, with a median of 11 (4). Despite the increase in symptom severity, the QoL score decreased in the 64 Gy/32 fraction group. However, as can be seen in Table 5, QoL scores remained higher in the 64 Gy/32 fraction group relative to the 36 Gy/6 fraction group.

**Table 5:** Follow-up questionnaires (12 months)

Follow-up 12 months	64 Gy/32 fraction group (*n*=4/7)	36 Gy/6 fraction group (*n*=2/9)
MLUTS total, median (IQR)	20 (6)	6 (2)
Voiding symptoms, median (IQR)	9 (2)	3 (0)
Storage symptoms, median (IQR)	11 (4)	3 (2)
QoL voiding, median, (IQR)	8 (16)	0 (0)
QoL storage, median, (IQR)	7 (18)	3 (3)
QoL total, median (IQR)	16 (35)	3 (3)

Abbreviations: IQR: Interquartile range; MLUTS: Male lower urinary tract symptom score; *n*: Number; QoL: Quality of life.

Bladder function diary results (Table 6) indicated that, among patients receiving 64Gy/32 fractions, 18 – 36% reported hematuria, peaking in week 3. Involuntary voiding was reported by 36% of patients in week 1. However, this percentage declined throughout the treatment period. About 50% of patients reported dysuria from week 1 onward, with the incidence increasing to 80% by week 6. In comparison, hematuria was reported by 10 – 33% of patients in the 36 Gy/6 fraction group, peaking in week 5. Involuntary voiding remained low in this group, with a maximum of 20% reporting symptoms in weeks 1 and 4. Dysuria was reported by only 10% of patients in this group in week 1; however, the incidence increased to 56% by week 6.

**Table 6:** Bladder function diary throughout radiation therapy

Detail	Week 1		Week 2		Week 3		Week 4		Week 5		Week 6	
					
	A	B	A	B	A	B	A	B	A	B	A	B
Catheter, *n* (%)	3 (27)	4 (40)	3 (27)	4 (33)	3 (27)	5 (45)	4 (36)	5 (50)	3 (27)	5 (45)	3 (30)	3 (33)
Cystitis, *n* (%)	1 (9)	0 (0)	0 (0)	0 (0)	2 (18)	1 (9)	1 (9)	0 (0)	0 (0)	1 (9)	4 (40)	0 (0)
Hematuria, *n* (%)	2 (18)	1 (10)	2 (18)	2 (17)	4 (36)	3 (27)	3 (27)	3 (30)	2 (18)	4 (33)	2 (20)	1 (11)
Involuntary Void, *n* (%)	4 (36)	2 (20)	2 (18)	1 (8)	3 (27)	0 (0)	2 (18)	2 (20)	1 (9)	1 (9)	1 (9)	0 (0)
Dysuria, n (%)	6 (55)	1 (10)	6 (55)	5 (42)	7 (64)	4 (36)	5 (45)	5 (50)	7 (64)	6 (55)	8 (80)	5 (56)

Notes: A: 64 Gy/32 fraction group (11 patients); B: 36 Gy/6 fraction group (10 patients). Abbreviation: *n*: Number.

## 4. Discussion

RT is a well-established treatment modality for cancer. However, its adverse effects are well-documented and tend to significantly impact patients’ QoL. This study presented descriptive data from 23 patients treated with RT for BC, though the small sample size restricts the generalizability of the findings.

Although the Charlson comorbidity index (CCI) scores did not increase in patients receiving 36Gy/6 fractions, patients in this group were older than those receiving 64Gy/32 fractions, with a median age of 85 and 78 years, respectively. In addition, patients in the 36 Gy/6 fraction group reported a greater baseline impact of urinary symptoms on QoL than those in the 64 Gy/32 fraction group, despite similar baseline LUTS scores. There is no clear explanation for why patients in the 36 Gy/6 fraction group reported a greater impact of LUTS on their QoL. However, one possible explanation is that these patients were allocated to a treatment regimen considered less invasive, suggesting they may have been in poorer overall health than could be classified by the CCI.

Tran *et al*.^17^ investigated a cohort of 39 patients (median age: 78 years) who underwent RT as they were either unsuitable for surgery or had refused cystectomy. Their findings identified good performance status and younger age as favorable prognostic factors. In our study, the increased impact of urinary symptoms on QoL in the 36 Gy/6 fraction group was primarily associated with storage symptoms. The underlying reason for this higher score is unclear, though it may be related to age-associated mobility limitations, which exacerbate the inconvenience of urgency and increased frequency.

During treatment, LUTS scores in the 36 Gy/6 fraction group remained stable or even improved. Notably, although these patients experienced a substantial impact on QoL at baseline, this impact seemed to diminish over time. In contrast, patients in the 64 Gy/32 fraction group reported a higher LUTS-related QoL impact at the 4-month follow-up compared to baseline, most likely due to ARC.

During treatment weeks, a few patients reported hematuria. Dysuria was also observed throughout the treatment course in both groups, with a trend toward worsening symptoms during treatment. However, involuntary voiding symptoms appeared more prevalent in the 64 Gy/32 fraction group and were most pronounced in the initial weeks of treatment.

Radiation cystitis is predominantly characterized by storage-related symptoms^8^. However, in this study, we did not observe a higher score of storage LUTS compared to voiding LUTS, regardless of treatment group or follow-up time point. Furthermore, our data suggest that the total dose exerts a greater impact on LUTS and QoL than the dose per fraction.

As previously mentioned, the optimal RT protocol that balances efficacy with minimal side effects has been extensively investigated in the literature. While the standard treatment protocol for BC consists of 64 Gy in 32 fractions, an alternative approach has been proposed: hypofractionated RT with 55 Gy in 20 fractions over 4 weeks.^18^,^19^ In addition, for MIBC, trimodality treatment is emerging as a viable therapeutic option to cystectomy. Compared to RT alone, the combination of RT with concomitant radiosensitizing chemotherapy has demonstrated improved locoregional control.^19^ In addition to the growing awareness of systemic treatments administered concomitantly with RT, there are also ongoing technical advancements, which may further enhance RT efficacy without increasing side effects. A study by Hafeez *et al*.^20^ evaluated the maximum tolerated bladder tumor-focused dose in 59 patients, suggesting dose escalation to 70 Gy is feasible with acceptable toxicity and a bladder preservation rate of 89%.

LUTS are influenced by multiple factors, making it difficult to draw definitive conclusions from small study populations. However, higher radiation doses may exacerbate symptoms, thereby negatively affecting patients’ QoL. In turn, a compromised QoL may contribute to reluctance in administering the full regimen of 32 RT fractions.

Following cancer treatment, particularly RT, post-treatment surveillance is essential for detecting recurrences, functional impairments, or other treatment-related side effects. However, evidence guiding the optimal surveillance plan and follow-up remains sparse and requires further investigation to adequately address the late effects of cancer treatment.^21^ Long-term side effects of RT often present years after treatment completion. In recent years, advancements in treatment techniques and diagnostic measures have led to increased cancer survivorship, rendering patient QoL an increasingly important focus.^22^

Despite a 5-year post-treatment bladder preservation rate of 60 – 87%,^21^ pelvic radiation can result in tissue injuries, leaving patients to contend not only with the cancer but also with treatment-related side effects. Although RT is initially intended as a curative approach, its late effects are strongly associated with psychological distress, potentially leading to a reduced QoL among survivors. Addressing late effects should therefore extend beyond physical complications to include psychological aspects, as impaired QoL has been linked to increased mortality and greater consumption of medical resources.^23^ Various strategies have been explored to mitigate RT-induced late effects, leading to advancements in treatment delivery systems and planning. These innovations integrate multiple tools to minimize excess radiation exposure to adjacent organs.^24^ Nevertheless, before initiation of RT, patients should be informed of potential treatment-related consequences and symptoms to monitor. Systemic symptom evaluation should be a routine part of follow-up care, ensuring early detection and timely management of persistent symptoms.^23^

The combination of chemotherapy and RT has been shown to improve BC-specific survival and local tumor control. In a large randomized controlled trial on bladder-sparing treatment for MIBC, Huddart *et al*.^25^ reported that chemotherapy combined with RT did not negatively affect QoL outcomes. Notably, while patients experienced an initial decline in QoL following treatment, their scores returned to baseline levels within 6 months.

As modern medicine increasingly embraces a holistic approach to patient care, the psychological side effects of treatment, quantified as QoL, among patients treated for BC or suffering from radiation cystitis has increasingly become a focus of healthcare professionals and patients.^22^ QoL is a multidimensional concept encompassing both subjective and objective aspects from the patient’s perspective. Chronic conditions, such as radiation cystitis, are known to negatively affect patients’ QoL. A study by Rapariz-González *et al*.^26^ investigated the impact of urinary symptoms on patients diagnosed with painful bladder/pelvic pain syndrome and radiation cystitis. They assessed symptom severity and QoL impairment using the pelvic pain and urgency/frequency score and evaluated self-esteem and anxiety levels on Rosenberg’s self-esteem scale and Goldberg’s anxiety scale. Their findings indicated that patients with these conditions experienced significant declines in self-esteem and increased anxiety compared to the general population. Notably, they concluded that the impact of radiation cystitis on men’s QoL was comparable to the negative effects of erectile dysfunction.^26^

For cancer survivors, another critical aspect of QoL is the need for individualized evaluation of potential treatment-related side effects. A patient-centered approach allows for a more comprehensive understanding of treatment-related impacts.^25^

The management of radiation cystitis primarily focuses on identifying or excluding other causes of urinary symptoms, especially urinary tract infections, and cancer recurrence. Treatment is tailored to symptom severity. During the acute phase of cystitis, hematuria is a major symptom and is managed with bladder irrigation, clot evacuation, and, in certain cases, fluid resuscitation. Once the patient is stabilized, additional treatment modalities can be introduced.^27^ These modalities include systemic therapies such as anticholinergic medications, β_3_-adrenergic receptor agonists, and analgesics. Symptomatic treatments may also involve bladder irrigation with hyaluronic acid to restore the glycosaminoglycan layer or, in some cases, surgical intervention to evacuate hemorrhagic clots.^8^,^9^ Hyperbaric oxygen therapy is another treatment modality in which the patient breathes 100% pure oxygen for 90 min at a pressure equivalent to 14 meters below sea level, 5 times per week for 6 – 8 weeks. This therapy promotes angiogenesis and healthy granulation tissue formation, thereby enhancing oxygen delivery to damaged tissues. In addition, it induces vasoconstriction, which helps alleviate both inflammatory and hemorrhagic symptoms.^27^,^28^ A randomized controlled phase II – III trial evaluating hyperbaric oxygen therapy for radiation cystitis was conducted across five Nordic university hospitals, enrolling a total of 82 patients. The treatment was well tolerated and demonstrated a favorable safety profile, with data suggesting symptomatic relief in patients with late-onset radiation cystitis.^28^

In addition to managing radiation-induced side effects, efforts have been directed toward their prevention. A randomized controlled trial by Redorta *et al*.^29^ evaluated the prophylactic use of hyaluronic acid in two forms: Intravesical instillations and oral formulations, before RT. The study utilized the Expanded Prostate Cancer Index Composite, the ICIQ-MLUTS, and the EuroQol Group EQ-5D-5L for evaluation. Results indicated that the intervention group had improved questionnaire scores related to urinary tract symptoms compared to patients who did not receive the pre-radiation treatment. Building on this, researchers are also investigating other preventative approaches, including stem cell therapy.^27^

Currently, no consensus has been reached on predicting which patients will develop radiation cystitis following treatment. Variability in radiation protocols and symptom presentations further complicates the establishment of standardized guidelines for identifying at-risk patients.^14^

The present study has several limitations; the most notable is the low inclusion rate. Although the project was conducted across four different sites in Denmark over a 3-year period, the inclusion rate remained low. This low rate may be attributed to the frailty of the patient population, the psychological burden of a cancer diagnosis, and the logistical challenges associated with multiple radiation fractions. These factors likely contributed to some patients declining participation. In addition, in Denmark, radical cystectomy remains the preferred treatment for BC, which may have further influenced enrollment.

Another limitation was the difficulties in accurately recording urinary symptoms, as many patients had a catheter placed at some point during the study period. Consequently, symptoms related to radiation cystitis could not be adequately assessed within the scope of the questionnaire. In addition, urinary symptom data were collected through telephone interviews, making responses susceptible to acquiescence and social desirability bias, thereby compromising the reliability of self-reported outcomes. Patients asked to recall urinary symptoms before catheter placement were also at risk of recall bias. During the final follow-up, additional challenges arose in obtaining responses to the final questionnaire. Some patients were deceased, others had catheter placements, some declined further participation, and others could not be reached. These difficulties also prevented the inclusion of data from frequency-volume charts, as many were incomplete or inconsistently filled out.

Due to the low patient inclusion rate, modifications to the original study protocol were necessary. The initial protocol included a patient group undergoing androgen deprivation therapy for prostate cancer to evaluate the potential effect of hormone depletion during RT. Given the challenges in treating radiation cystitis, strategies for its prevention or reversal could be valuable. Inhibition of sex steroids through the use of luteinizing hormone-releasing hormone antagonists or agonists has been proposed as a potential radioprotective approach, as it may mitigate normal tissue damage and promote hematopoietic regeneration.^30^ However, only two patients diagnosed as having prostate cancer before a BC diagnosis were enrolled, and among them, only one received androgen deprivation therapy. Furthermore, the original follow-up protocol included two urodynamic examinations to evaluate the functional consequences of RT on the detrusor muscle and a pathological assessment of bladder fibrosis. However, patients were generally reluctant to make additional hospital visits and showed little interest in these examinations, preventing data collection in these areas. Similarly, due to the low inclusion rate, no meaningful conclusions could be drawn from the limited bladder pathology specimens harvested.

## 5. Conclusion

Due to the low inclusion rate, drawing definitive conclusions regarding differences in side effects between the two RT strategies remains difficult. However, patients who received the 64 Gy/32 fraction regimens were more likely to experience symptoms both during treatment and in the follow-up periods. This finding suggests that the total dose may have a greater impact on RT-related side effects than the dose per fraction.

## Data Availability

Data can be made available to readers from the corresponding author upon request.
